# Analysis of the susceptibility of lung cancer patients to SARS-CoV-2 infection

**DOI:** 10.1186/s12943-020-01209-2

**Published:** 2020-04-28

**Authors:** Qi Kong, Zhiguang Xiang, Yue Wu, Yu Gu, Jianguo Guo, Fei Geng

**Affiliations:** 1 Institute of Laboratory Animal Sciences, Chinese Academy of Medical Sciences (CAMS) and Comparative Medicine Center, Peking Union Medical College (PUMC), Key Laboratory of Human Disease Comparative Medicine, Chinese Ministry of Health; Beijing Key Laboratory for Animal Models of Emerging and Reemerging Infectious Diseases, 5 Panjiayuan Nanli, Chaoyang District, Beijing, 100021 P.R. China; 2 W Booth School of Engineering Practice and Technology, McMaster University, 1280 Main Street West, Hamilton, Ontario L8S 0A3 Canada

**Keywords:** SARS-CoV-2, COVID-19, TMPRSS2, ACE2, Lung Cancer

## Abstract

Recent studies have reported that COVID-19 patients with lung cancer have a higher risk of severe events than patients without cancer. In this study, we investigated the gene expression of angiotensin I-converting enzyme 2 (ACE2) and transmembrane serine protease 2 (TMPRSS2) with prognosis in lung adenocarcinoma (LUAD) and lung squamous cell carcinoma (LUSC). Lung cancer patients in each age stage, subtype, and pathological stage are susceptible to SARS-CoV-2 infection, except for the primitive subtype of LUSC. LUAD patients are more susceptible to SARS-CoV-2 infection than LUSC patients. The findings are unanimous on tissue expression in gene and protein levels.

Since Dec 2019, 2019 novel coronavirus disease (COVID-19) has emerged infecting with severe acute respiratory syndrome coronavirus 2 (SARS-CoV-2) and created a global epidemic with over 2.5 million patients in most countries of the world with more than 174 thousand deaths (updated at 20 April 2020). The coronavirus receptor angiotensin I-converting enzyme 2 (ACE2) and the activator transmembrane serine protease 2 (TMPRSS2) have been recognized as participants in SARS-CoV-2 host cell entry.

Patients with lung cancer are more susceptible to infection than normal individuals [1, 2]. Lung cancer is the most important malignant tumor with the first incidence rate and mortality rate in China, and even in the world. It is necessary to investigate the susceptibility of lung cancer to SARS-CoV-2 at molecular levels and take basic prevention methods. We investigated the expression of ACE2 and TMPRSS2 and their associations with prognosis in two common lung cancers, lung adenocarcinoma (LUAD) and lung squamous cell carcinoma (LUSC). We aim to explore the expression differences in ACE2 and TMPRSS2 between these lung cancers and their relationships with SARS-CoV-2 infection.

## Gene expression analysis of human lung cancer pathological stages and subtypes by GEPIA2

**Fig. 1:**
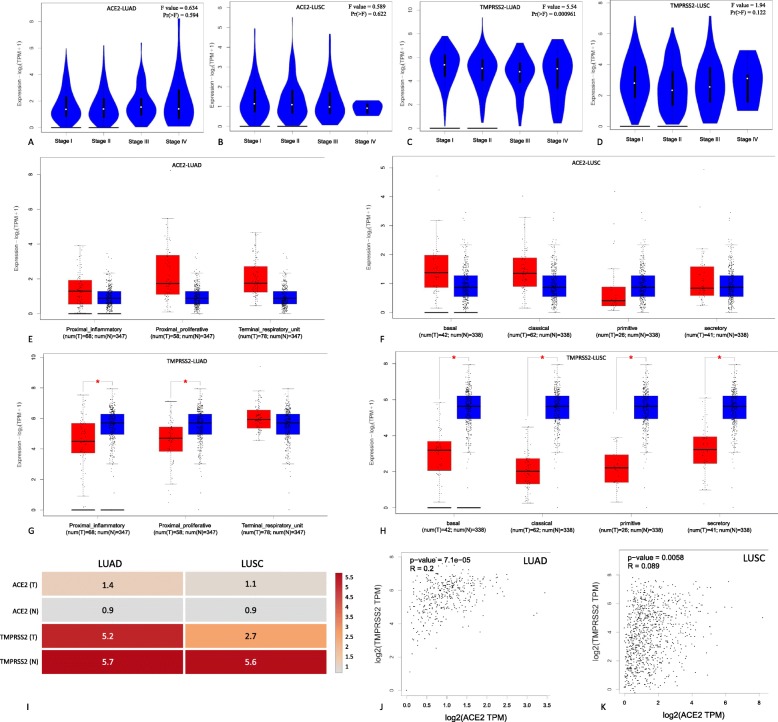
The expression level trends of the ACE2 and TMPRSS2 genes in lung tissues. **a** ACE2 gene expression level trends in different LUAD pathological stages; **b** ACE2 gene expression level trends in different LUSC pathological stages; **c** TMPRSS2 gene expression level trends in different LUAD pathological stages; **d** TMPRSS2 gene expression level trends in different LUSC pathological stages. **e** ACE2 gene expression in each subtype of LUAD; **f** ACE2 gene expression in each subtype of LUSC; **g** TMPRSS2 gene expression in each subtype of LUAD; **h** TMPRSS2 gene expression in each subtype of LUSC; **i** Comparison of ACE2 and TMPRSS2 gene expression levels in lung cancer tissues (T) and lung normal tissues (N); **j** ACE2 and TMPRSS2 gene co-expression in lung normal tissues (*p*-val = 7.1e^− 05^); and **k** ACE2 and TMPRSS2 gene co-expression in lung cancer tissues (*p*-val = 0.0058)

By using the Gene expression profiling interactive analysis 2 (GEPIA2), we profiled the expression of the ACE2 and TMPRSS2 genes in each pathological stage of two lung cancer types (LUAD and LUSC) using box plots. ACE2 gene expression in each pathological stage of LUAD was no different (*F*-val = 0.634, Fig. 1a); likewise, in LUSC, it was also no different (*F*-val = 0.589, Fig. 1b). TMPRSS2 gene expression in each pathological stage of LUAD showed obvious differences (*F*-val = 5.54, Fig. 1c), but in LUSC, it did not vary (*F*-val = 1.94, Fig. 1d). The data show that changes in the expression of these two genes had no statistical significance, which suggests that the ACE2 and TMPRSS2 genes are expressed consistently in each pathological stage of lung cancer, except for TMPRSS2 in LUAD. Furthermore, there are no significant differences in the susceptibility to SARS-CoV-2 among the pathological stages of LUAD and LUSC. TMPRSS2 maybe a cancer suppressor gene in LUAD, the downregulated expression level may decrease the susceptibility to SARS-CoV-2 for LUAD patients.

With gene expression data, previous studies have classified LUAD into three subtypes including terminal respiratory unit (TRU), proximal inflammatory (PI), proximal proliferative (PP) [3], and classified LUSC into four subtypes, including the primitive, classical, secretory and basal subtypes [4]. These subtypes have different survival outcomes and susceptible to drugs and viruses. We profiled the gene expression data for ACE2 and TMPRSS2 in LUAD, and LUSC subtypes.

ACE2 gene expression in each subtype of LUAD is all higher in lung cancers than normal lung tissues (Fig. 1e), especially in the proximal proliferative subtype. While in LUSC, they are similar, but the ACE2 gene expression level in the primitive subtype is higher in normal lung tissues than lung cancers (Fig. 1f). These data may show the susceptibility to SARS-CoV-2 among each subtype of LUAD and LUSC. In converse, TMPRSS2 gene expression in each subtype of LUAD is all lower in cancers than normal lung tissues, but little difference in terminal respiratory unit subtype (Fig. 1g). In LUSC, TMPRSS2 gene expression is much lower than LUAD (Fig. 1h).

We also profiled the tissue-specific expression of the ACE2 and TMPRSS2 genes in two lung cancer types (LUAD and LUSC) using an interactive heat map. The expression of the ACE2 and TMPRSS2 genes in different lung cancer stages is illustrated in Fig. 1i. The TMPRSS2 gene was expressed at higher levels in normal lung tissue than ACE2 and also more highly expressed in LUAD. We determined the ACE2 and TMPRSS2 gene coexpression in lung tissues. The ACE2 and TMPRSS2 genes were co-expressed in normal lung tissue (Fig. 1j, *p*-val = 7.1e^− 05^) and lung cancer tissue (Fig. 1k, *p*-val = 0.0058), but the differences were statistically significant (*p*-val < 0.01).

## Gene expression meta-analysis of human lung cancers by LCE

**Fig. 2:**
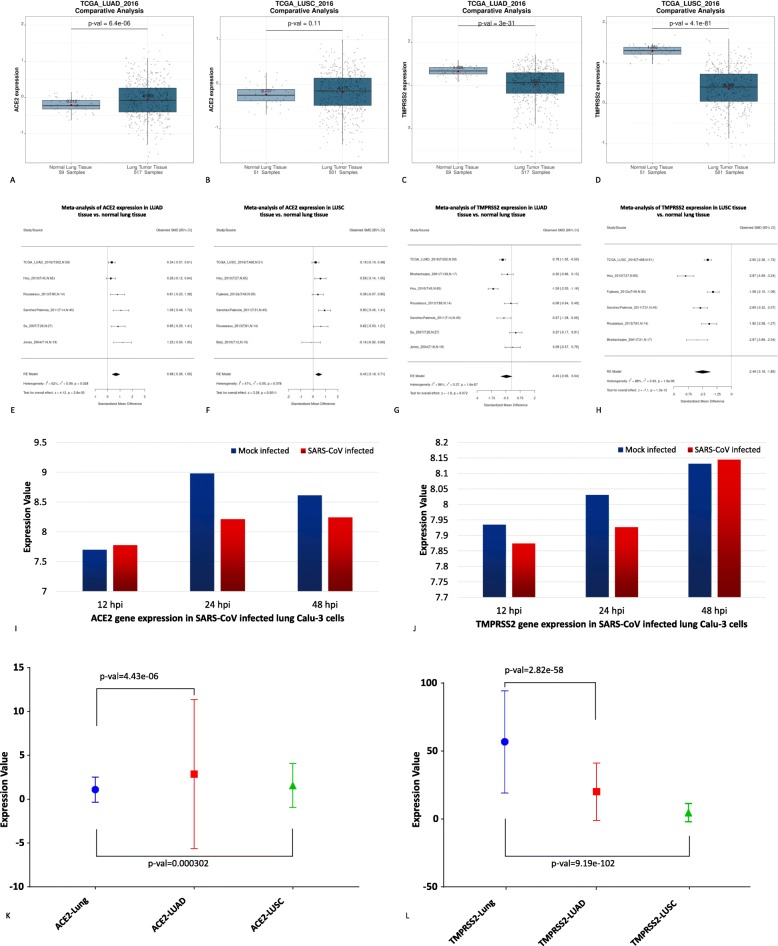
Comparative meta-analysis of the tissue-specific differential expression of the ACE2 and TMPRSS2 genes in lung tissues performed by LCE. **a** ACE2 in normal lung tissue and lung cancer tissue samples from TCGA_LUAD_2016 (*p*-val = 6.4e^− 06^); **b** ACE2 in normal lung tissue and lung cancer tissue samples from TCGA_LUSC_2016 (*p*-val = 0.11); **c** TMPRSS2 in normal lung tissue and lung cancer tissue samples from TCGA_LUAD_2016 (*p*-val = 3e^− 31^); **d** TMPRSS2 in normal lung tissue and lung cancer tissue samples from TCGA_LUSC_2016 (*p*-val = 4.1e^− 81^); **e** Meta-analysis of ACE2 expression in LUAD tissue vs. normal lung tissue(*p*-val = 3.8e^− 05^, *I*^2^ = 62%); **f** Meta-analysis of ACE2 expression in LUSC tissue vs. normal lung tissue (*p*-val = 0.0011, *I*^2^ = 47%); **g** Meta-analysis of TMPRSS2 expression in LUAD tissue vs. normal lung tissue (*p*-val = 0.072, *I*^2^ = 86%); **h** Meta-analysis of TMPRSS2 expression in LUSC tissue vs. normal lung tissue (*p*-val = 1.3e^− 12^, *I*^2^ = 88%); **i** ACE2 genes expression in human airway bronchial epithelial cells (Calu-3, a non-small-cell lung cancer cell line) infected with SARS-CoV; and **j**. TMPRSS2 genes expression in human airway bronchial epithelial cells (Calu-3, a non-small-cell lung cancer cell line) infected with SARS-CoV; **k** ACE2 protein expression among lung tissue, LUAD, and LUSC with data from HPA; **l** TMPRSS2 protein expression among lung tissue, LUAD, and LUSC with data from HPA

To further understand the implications of the differential expression of the ACE2 gene between normal lung tissue and cancerous lung tissue, we compared ACE2 gene expression between these two tissue types in LCE, and the results are shown in a box plot of the two selected groups. The data showed that the ACE2 gene was expressed at higher levels in LUAD cancer tissue than in normal lung tissue (Fig. 2a, *p*-val = 6.4e^− 06^) and the ACE2 gene was expressed at nearly equal levels in LUSC cancer tissue and normal lung tissue (Fig. 2b, *p*-val = 0.11). However, the data showed that the TMPRSS2 gene was expressed at higher levels in normal lung tissue than in LUAD cancer tissue (Fig. 2c, *p*-val = 3e^− 31^) or LUSC cancer tissue (Fig. 2d, *p*-val = 4.1e^− 81^).

Meta-analyses showed no significant correlation between ACE2 gene expression and LUSC (Fig. 2e, *I*^2^ = 47%, *p*-val = 0.0011) or between ACE2 gene expression and LUAD (Fig. 2f, *I*^2^ = 62%, *p*-val = 3.8e^− 05^). This suggests that ACE2 gene expression is not significantly different between normal lung tissue and cancerous lung tissue. TMPRSS2 gene expression had a significant association with negative expression outcomes in LUSC (Fig. 2g, *I*^2^ = 88%, *p*-val = 1.3e^− 12^); however, there was no significant association in LUAD (Fig. 2h, *I*^2^ = 86%, *p*-val = 0.072). This suggests that TMPRSS2 gene expression exhibits a meaningful difference between normal lung tissue and LUSC tissue but not between normal lung tissue and LUAD tissue.

## Gene expression analysis in SARS-CoV infected animal models and human lung epithelial cells

In SARS-CoV-2-infected hACE2 transgenic mice, the typical histopathology was interstitial pneumonia with significant inflammatory cell infiltration around the bronchioles and blood vessels, and viral antigens were observed in bronchial epithelial cells and alveolar epithelial cells [5]. SARS-CoV infected hACE2 transgenic mice had severe pulmonary lesions, including interstitial hyperemia and hemorrhage, monocytic and lymphocytic infiltration, protein exudation, and alveolar epithelial cell proliferation and desquamation [6]. SARS-CoV infected TMPRSS2-KO mice showed weakened inflammatory chemokine and/or cytokine responses to intranasal stimulation. TMPRSS2 deficiency affected the primary sites of infection and virus spread within the airways, which was accompanied by relatively less severe immunopathology [7].

The ACE2 and TMPRSS2 genes have obvious differential expression between SARS-CoV infected cells and Mock infected Calu-3 (subclone 2B4) cells, with *p*-val of 5.07e^− 12^ vs 4.65e^− 45^, respectively, in GSE17400, which demonstrated that the SARS-CoV induced secretion of cytokines by epithelial Calu-3 cells could exacerbate or dampen host inflammatory and T cell responses [8]. However, in the lung cancer samples in GSE19804, the difference in TMPRSS2 gene expression was not obvious (*p*-val = 2.37e^− 01^). ACE2 gene expression was more reduced in Calu-3 cells infected with SARS-CoV than in lung cancer samples when compared with TMPRSS2 expression in the average of 12, 24, 48 h post-infection (hpi), especially in 24 hpi (Fig. 2i-j).

Furthermore, the lung tissue expressions of ACE2 and TMPRSS2 in protein levels are exhibited with data from human protein atlas (HPA). The ACE2 protein was expressed at higher levels in LUAD cancer tissue (*p*-val = 4.43e^− 06^) and LUSC (*p*-val = 0.000302) than in normal lung tissue, with significance difference among normal lung, LUAD, and LUSC (Fig. 2k, *p*-val < 0.001). TMPRSS2 protein was expressed at decreased levels accordingly among normal lung, LUAD, and LUSC (Fig. 2l, *p*-val < 0.001). These data are unanimous to the tissue expression in gene level.

## Conclusions

The gene expression level of ACE2 may indicate the susceptible to SARS-CoV-2 infection, and TMPRSS2 plays a supporting role. We reported that the comparative gene expression of ACE2 and TMPRSS2 in each subtype and pathological stages of two common lung cancers, LUAD and LUSC. Lung cancer patients in each age stage, subtype, and pathological stages are susceptible to SARS-CoV-2 infection, except for the primitive subtype of LUSC, and were verified by meta-analysis, gene expression omnibus (GEO) data and animal models results. Besides, TMPRSS2 maybe a cancer suppressor gene for it was severely downregulated in LUAD and LUSC and the mechanism was not reported yet.

## Data Availability

Not applicable.

## Abbreviations

ACE2: Angiotensin I converting enzyme 2
COVID-19: 2019 novel coronavirus disease
GEPIA2: Gene expression profiling interactive analysis 2
HPA: Human protein atlas
LCE: Lung cancer explorer
LUAD: Lung adenocarcinoma
LUSC: Lung squamous cell carcinoma
SARS-CoV-2: Severe acute respiratory syndrome coronavirus 2
TMPRSS2: Transmembrane serine protease 2
